# Metabolomic Analysis Provides Insights into Bud Paradormancy in *Camellia sinensis* cv. Huangdan

**DOI:** 10.3390/ijms26115094

**Published:** 2025-05-26

**Authors:** Mingjie Chen, Zhenghua Du, Wenjie Yue, Xiangrui Kong, Quanming Xu, Dongsheng Fang, Changsong Chen

**Affiliations:** 1 College of Life Sciences, Xinyang Normal University, Xinyang 464000, China; fangdongsheng@xynu.edu.cn; 2 Tea Research Institute, Fujian Academy of Agricultural Sciences, The Fujian Research Branch of the National Center for Tea Genetic Improvement, Fuzhou 350012, China; kongxiangrui@faas.cn; 3 Fujian Provincial Key Laboratory of Haixia Applied Plant Systems Biology, Haixia Institute of Science and Technology and School of Life Sciences, Fujian Agriculture and Forestry University, Fuzhou 350002, China; zhenghuadu@fafu.edu.cn (Z.D.); xquanming@fjpsc.edu.cn (Q.X.); 4 Jinshan College, Fujian Agriculture and Forestry University, Fuzhou 350002, China; wj_yue07@fafu.edu.cn

**Keywords:** axillary floral bud, axillary shoot bud, *Camellia sinensis*, metabolomics, paradormancy, sugars, flavonoids

## Abstract

Bud paradormancy has been widely studied in perennial deciduous woody species, but little attention has been paid to paradormancy set and release in perennial evergreen tree species. Here, shoot bud paradormancy in *Camellia sinensis* cv. Huangdan was studied by untargeted metabolomics. We found that after removing the axillary floral buds for one day, the paradormancy of the axillary shoot buds was released. The paradormant shoot buds had lower glucose-1-phosphate, fructose, and D-(-)-tagatofuranose content but higher trehalose, raffinose, galactinol, and α-D-xylopyranose content. Meanwhile, high levels of asparagine were accumulated. Flavonoids were differentially accumulated, and higher levels of three flavone glycosides (C-diglucosylapigenin, apigenin 6-C-glucoside 8-C-arabinoside, and prunin) and four proanthocyanidins (Procyanidin trimer isomer 1, Galloylprocyanidin dimer, Procyanidin trimer isomer 3, and Galloylated trimeric proanthocyanidin) were accumulated in paradormant shoot buds. During the paradormancy-to-growth transition, all these metabolites were reversed. These data suggest that the reconfiguration of carbon, nitrogen, and flavonoid metabolism could be an important aspect for the paradormancy set and release of tea axillary shoot buds. This study provided novel insights into shoot bud paradormancy set and release in a perennial evergreen tree species.

## 1. Introduction

In angiosperms, the axillary buds are formed from the axillary meristems initiated at the base of emerging leaves, and they contain preformed shoot meristems and leaf primordia [1]. Under certain conditions, the axillary buds can enter into dormancy rather than growing out immediately. Both environmental and endogenous factors could induce bud dormancy set and release, including nutrient or water availability, temperature, light quality, day length, sink and source activities, hormone levels, and the epigenetic modification of growth-related genes [2,3,4,5]. Based on the types of stimuli that promote bud growth arrest, bud dormancy can be divided into paradormancy, endodormancy, and ecodormancy [6]. Paradormancy is a temporary bud growth suspension caused by biochemical signals from other organs or tissues.

Metabolic reconfiguration is closely correlated with bud dormancy. For example, decapitation in hybrid aspen induces the paradormancy release of axillary buds; furthermore, gibberellin and plasmodesmata communication play important roles during the paradormancy-to-growth transition [7], while early defoliation in pear also promotes floral bud paradormancy release, a process which auxin efflux is involved in [8]. The endodormancy release of leaf spurge crown buds, almond flower buds, and poplar apical buds is accompanied by the metabolite reconfiguration of sugars, sugar alcohols, amino acids, organic acids, and flavonoids [9,10,11]. So far, bud dormancy has primarily been studied in perennial deciduous woody or herbaceous species [12,13,14,15]. These investigations have suggested that carbon starvation could be the major cause of bud dormancy induction [16], and trehalose-6-phosphate (Tre6P) acts as a proxy for carbon status, participating in sugar sensing [17].

As of now, few studies have been conducted on perennial evergreen woody species. As a result, whether evergreen trees and deciduous ones share similar dormancy regulation mechanisms remains an open question. As a perennial evergreen woody tree species, *Camellia sinensis* shows ‘Banjhi dormancy’ and winter dormancy [2,5,18]. ‘Banjhi dormancy’ is a form of endogenous rhythmic growth and bud growth arrest under favorable conditions, which usually takes place several times during a growing season [18], while winter dormancy occurs in all tea plants growing beyond 16° N or S latitudes when the day length is shorter than 11 h during winter [2,5]. Sugars are involved in tea bud cold acclimation and winter dormancy [19,20,21].

In this study, *Camellia sinensis* cv. Huangdan is used as a model perennial evergreen tree species to study paradormancy set and release. The axillary floral buds were removed first, and then the axillary shoot buds were harvested on the first and third days after floral bud removal (HD-1 and HD_3). On the day of removing the local axillary floral buds, some of the axillary shoot buds also were harvested as control (HD_0 samples); untargeted metabolomic analysis was then performed to characterize the effects of the axillary floral bud removal on local shoot bud metabolome. This study offers new insights into metabolomic reconfigurations during paradormancy-to-growth transitions in tea axillary shoot buds.

## 2. Results

### 2.1. The Axillary Shoot Bud Metabolome Was Significantly Modified One Day After Removing the Local Axillary Floral Buds

**Figure 1:**
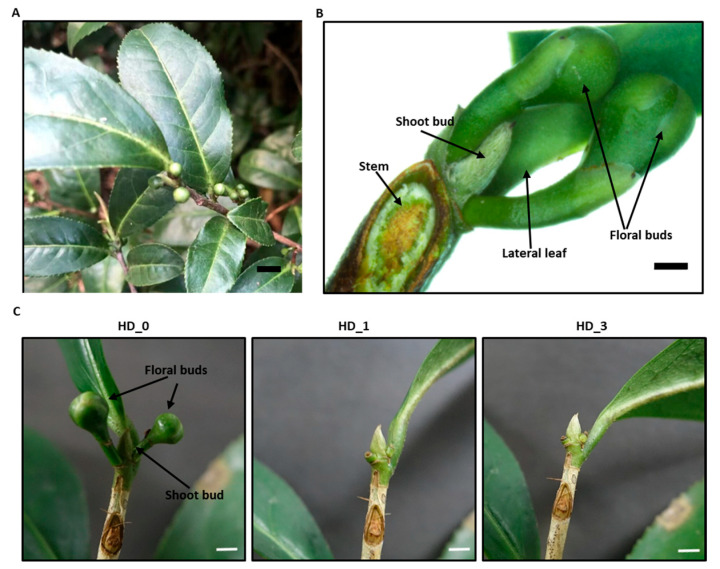
Photography of *Camellia sinensis* cv. Huangdan. (**A**) *Camellia sinensis* cv. Huangdan grown in the tea garden, bar = 3 mm; (**B**) a close-up view showing the arrangement of the auxiliary shoot bud, the auxiliary floral buds and the lateral leaf which are clustered at the same leaf node, the stem above the node was removed for photography, bar = 2 mm. (**C**) The same axillary shoot bud before (HD_0) and after removing the axillary floral buds for 1 day (HD_1) and 3 days (HD_3), bar = 5 mm.

**Figure 2:**
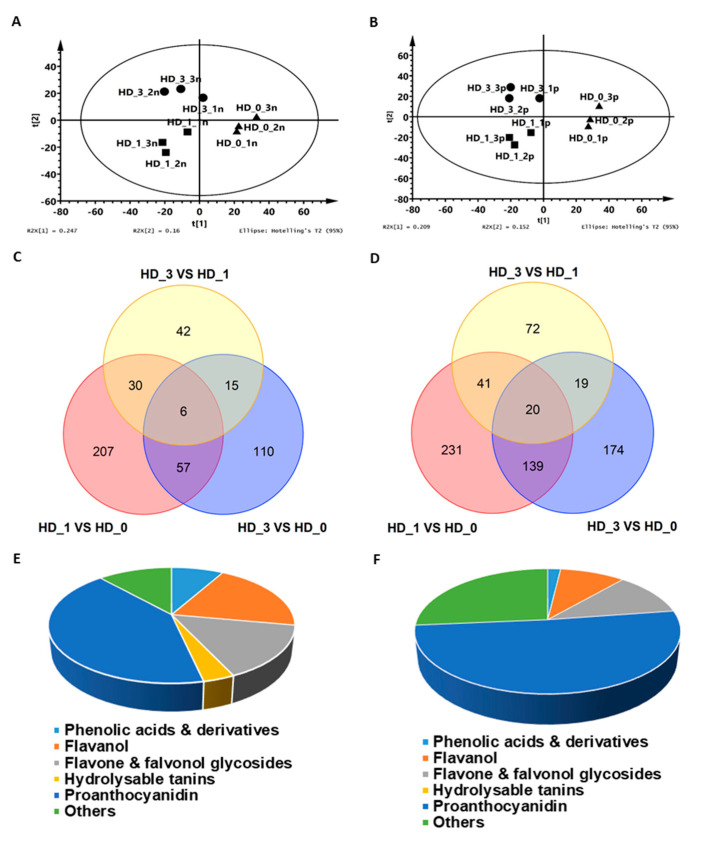
Partial least square-discriminant analysis (PLS-DA) and Venn diagram analysis of metabolome data obtained on LC-MS platform. PLS-DA results from the negative ionization mode (**A**) and the positive ionization mode (**B**), respectively. Venn diagram results from the negative ionization mode (**C**) and the positive ionization mode (**D**), respectively. Classification and distribution of metabolites chemically identified in the negative mode (**E**) and positive ion mode (**F**), respectively. HD_0, HD_1, and HD_3 represent before and after 1 and 3 days of the axillary floral bud removal, respectively.

In *Camellia sinensis* cv. Huangdan, the axillary floral buds and axillary shoot buds both emerged from the base of lateral leaves simultaneously in August (Figure 1A). The axillary shoot buds are located at the center and enclosed by two axillary floral buds (Figure 1B). The axillary floral buds grow normally, while the growth of axillary shoot buds is arrested (Figure 1A,B). To better understand this developmental pattern, the axillary floral buds were manually removed, while the local axillary shoot bud was kept intact. After the respective axillary floral buds were removed for 1 and 3 days, the axillary shoot buds were then harvested. During this period, the axillary shoot buds did not show obvious growth (Figure 1C). The shoot bud samples were extensively extracted, and the metabolites were analyzed by UPLC-QTOF MS (Supplementary Figure S1). In total, 2063 and 3288 metabolic tags were detected from the negative ionization mode and the positive ionization mode, and 467 and 696 of them showed differential abundance (*p* < 0.05), respectively (Supplementary Data S1; Supplementary Data S2). PLS-DA analysis revealed that the X-component explained 20.9–24.7% of the variance and clearly separated HD_0 from HD_1 and HD_3, while the Y-component explained 15.2–16% of the variance (Figure 2A,B), suggesting that the metabolic difference between the HD_1 and HD_3 samples was smaller compared to the HD_0 samples. Our results indicate that these three sample groups were in divergent metabolic states. Statistical analyses were performed to compare metabolite changes before and after the axillary floral bud removal. Under negative ionization mode, 300, 93 and 188 metabolite tags showed differential abundance for the HD_1 vs. HD_0, HD_3 vs. HD_1 and HD_3 vs. HD_0 comparison. Under positive ionization mode, 431, 152 and 352 metabolite tags showed differential abundance for the same set of comparisons. The distribution of the number of metabolite tags that are unique and common among these three sets of comparisons is presented in a Venn diagram (Figure 2C,D). Under the negative ionization mode, 207 out of 300 metabolites with differential abundance were unique for HD_1 vs. HD_0, 42 out of 93 were unique for HD_3 vs. HD_1, and 110 out of 188 were unique for HD_3 vs. HD_0 (Figure 2C). Under the positive ionization mode, 231 out of 431 metabolites with differential abundance were unique for HD_1 vs. HD_0, 72 out of 152 were unique for HD_3 vs. HD_1, and 174 out of 352 were unique for HD_3 vs. HD_0 (Figure 2D). These data demonstrated that 1 d after the floral bud removal (DAF), the axillary shoot buds experienced the most dramatic metabolic reconfiguration, which could be an indication of the paradormancy release at this time point.

By querying with an in-house authentic standard library and other online sources, 86 and 54 metabolites were chemically identified from the negative ionization mode and the positive ionization mode, respectively (Supplementary Data S3; Supplementary Data S4). Alkaloids were detected only from the positive ionization mode. The identification of some secondary metabolites was affected by the applied ionization modes (Figure 2E,F). One-way ANOVA analysis was performed to identify metabolites with significant changes. Under the negative ionization mode, 16 out of the 86 chemically identified metabolites showed significant changes; under the positive ionization mode, 12 out of 54 chemically identified metabolites showed significant changes.

To increase the coverage of identified metabolites, the axillary shoot bud samples were also analyzed by the GC-TOF MS platform (Supplementary Figure S2); 394 metabolic tags were detected (Supplementary Data S5), and 146 metabolites were chemically identified (Supplementary Data S6). One-way ANOVA analysis was applied, and 21 out of these 146 known metabolites were significantly altered. In total, 48 known metabolites with significant changes were uncovered; they are listed in Table 1. Several metabolites were identified by both ionization modes; in a such case, both results are presented. It worth noting that these metabolites showed similar changing trends, indicating the good reproducibility and reliability of these LC-MS results.

### 2.2. Sugars and Sugar Alcohols

Sugars and sugar alcohols are the major carbon source to fuel cell metabolism. In this study, seven sugars and sugar alcohols were significantly altered after paradormancy release. Three reducing hexoses (glucose-1-phosphate, fructose 1 and D-(-)tagatofuranose) showed lower contents in the paradormant shoot buds, then increased significantly at 1 DAF. In contrast, four non-reducing sugars and sugar alcohols (trehalose, raffinose, galactinol, and α-D-Xylopyranose) showed the highest contents in the paradormant shoot buds, and significantly decreased at 1 DAF (Table 1). Trehalose, raffinose, and galactinol are readily interconvertible with reducing hexoses [22,23,24,25]; this could explain why the increase in G-1-P and fructose at 1 DAF was accompanied by the concurrent decrease in trehalose, raffinose, and galactinol (Table 1). G-1-P and fructose are substrates for glycolysis; their lower contents in HD_0 samples suggest restricted carbon availability for respiration regardless of the abundant presence of non-reducing di- or tri- sacchrides such as trehalose, raffinose, and galactinol. α-D-xylopyranose is involved in cell wall synthesis; its higher content could be associated with reduced cell wall synthesis or elevated cell wall degradation in paradormant shoot buds. Besides acting as reserve carbohydrates, trehalose, raffinose, and galactinol could play other protective roles. For example, trehalose can act as a cryoprotectant, protein and membrane stabilizer and antioxidant [22]. Its phosphate form, trehalose-6-phosphate (Tre6P), plays a central role in the coordination of metabolism with development and stress response [17]. Raffinose (RFO) and galactinol can scavenge hydroxyl radicals to protect plant cells from oxidative damage [23], and can also act as membrane stabilizers, stress tolerance mediators, osmoregulators and mobile oligosaccharide signaling molecules [24,25].

### 2.3. Amino Acids and Amins

In this study, we found that asparagine (Asn) showed the highest contents in HD_0 samples, then gradually decreased in HD_1 and HD_3 samples. Meanwhile, two amins (N,N,O-Triacetylhydroxylamine and Benzenamine) were increased following paradormancy release (Table 1). Asparagine and some amines are naturally occurring osmolytes [26]; a higher Asn level not only serves as nitrogen storage but also facilitates paradormant shoot buds’ adaption to osmotic stress and prevents cellular protein structural changes. Dhuli et al. (2014) reported that following forced conifer bud break, Asn content was significantly increased [27], which is in sharp contrast with tea shoot buds (Table 1). Thus, even though both are perennial evergreen tree species, coniferous trees and broad-leaved tree buds may have different forms of nitrogen storage.

### 2.4. Organic Acids and Phenolic Acids

Six organic acids and phenolic acids showed lower contents in the paradormant shoot buds, then increased significantly at 1 or 3 DAF (Table 1). Arabinonic acid is derived from sugar oxidation [28]; its lower levels in the paradormant shoot buds is consistent with reduced sugar availability. Malonic acid provides carbon to form the A ring of flavonoids; thus, it is essential to transform phenolic acid into flavonoids (Figure S3). The lower levels of malonic acid (Table 1) suggest flavonoid synthesis was restricted in paradormant shoot buds. *P*-coumaroyl quinate is synthesized through quinate shunts, which use phenolic acids (quinate and *p*-coumaroyl-CoA) as substrates (Figure S3). The lower levels of *p*-coumaroyl quinate in paradormant shoot buds (Table 1) are likely associated with reduced supply of its precursors. The lower levels of malonic acid and phenolic acids (5-*p*-coumaroylquinic acid and benzoic acid) (Table 1) suggest that flavone synthesis in paradormant shoot buds would be reduced (Figure S3).

### 2.5. Flavones and Flavone Glycosides

One flavone (tricetin) and three flavone glycosides (C-diglucosylapigenin, apigenin 6-C-glucoside 8-C-arabinoside, and prunin) were identified with significant changes; they showed opposite changing trends. Tricetin showed lower levels in the paradormant shoot buds, and increased following paradormancy release. In contrast, the flavone glycosides showed higher levels in the paradormant shoot buds, then significantly decreased following paradormancy release at 1 DAF (Table 1). Since naringenin is the common precursor for the synthesis of flavone and flavone glycosides (Figure S3), these opposite changing patterns suggest that flavone supply was limited, while the flavone glycosylation was enhanced in paradormant shoot buds (Table 1).

### 2.6. Flavonol Glycosides and Falvanols

Two flavonol glycosides (Rutin, Quercetin 3-O-glucosyl rutinoside) and three flavanols (Gallocatechin 3′-O-gallate, Epigallocatechin gallate, and Epigallocatechin) were lower in the paradormant shoot buds, and significantly increased following paradormancy release (Table 1). This change pattern is consistent with that of phenolic acids, but opposite to flavone glycosides, as we described above.

### 2.7. Proanthocyanidins

Ten proanthocyanidins (PAs) were identified with significant changes; the majority of them showed higher content in the paradormant shoot buds, followed by a decrease after paradormancy release (Table 1). PAs are derived from flavanol oligomerization [29]; this may explain why higher PA levels in HD_0 samples were accompanied by a concurrent lower level of flavanol (Table 1). PA polymerization can sequestrate reactive pathway intermediates. In addition, PAs themselves possess protective functions against oxidative stress [29].

### 2.8. Antioxidants

α-Tocopherol and theobromine are potent antioxidants; their contents were lower in paradormant shoot buds, then increased significantly following paradormancy release (Table 1).

## 3. Discussion

Previous transcriptomic studies in perennial herbaceous and deciduous woody species suggest that carbon starvation is the initial trigger inducing bud dormancy [16,30,31]. In perennial evergreen woody tree species such as *Camellia sinensis* cv. Huangdan, the G-1-P and fructose contents in the paradormant shoot buds were the lowest, and significantly increased at 1 DAF (Table 1). The conifer bud break in evergreen Norway spruce and European silver fir can be induced by warm temperature treatment; the authors reported that glucose-6-phosphate (G6P) and fructose-6-phosphate (F6P) contents were increased [27]. These data are in accordance with the notion that carbon starvation could be a common factor for bud dormancy induction, regardless of whether they are deciduous or evergreen tree species. Even so, sugar profiles still could be affected by dormancy types, plant species, and bud types. For example, in short-day-induced poplar apical bud dormancy set, hexoses, including G6P, F6P, glucose, fructose, xylose, and maltose, exhibited lower levels, while raffinose and sucrose exhibited higher levels [9]; in contrast, in short-day-induced grapevine endodormancy, no differences in glucose, fructose, and sucrose concentrations were observed, while raffinose and trehalose levels were increased [32]. In almond flower bud endodormancy release, D-glucose exhibited a dramatic drop rather than an increase. Meanwhile, D-sorbitol-6-phosphate, D-fructose-2,6-biphosphate, and D-fructose-2-phosphate experienced a huge increase [11]. In paradormant crown bud of leaf spurge, both sucrose and hexose levels were the lowest, and increased during the transition into endodormancy [10].

Since reducing hexoses are the direct substrates for glycolysis and respiration, their reduced pool size would reduce ATP generation, leading to lower energy status; this may explain how shoot bud paradormancy is induced by the local growing floral buds (Figure 1). Considering that more carbons were stored in non-reducing oligomers such as trehalose, raffinose, and galactinol in paradormant shoot buds (Table 1), this suggests that the shoot buds are not passively adapting to carbon shortage. Instead, they actively reallocate available carbon sources into metabolically convenient forms for storage and protection. The accumulation of Asn (Table 1), a nitrogen-rich amino acid, not only serves as nitrogen storage and dehydration tolerance, but also helps to maintain the C/N balance in the paradormant shoot buds [26]. It also remains possible that shoot bud paradormancy is a sort of “altruistic” behavior by restricting its own nutritional demand, thus making more carbon source available to the growing floral buds and reproductive success.

Flavonoid accumulation is part of the adaptive response for plants [9,33]. They mitigate photooxidative damage of the organs present in buds by acting as a UV screener [34]. In almond flower buds, anthocyanins and flavonol glycoside was involved in endodormancy release [11]. In apricot floral buds, the contents for six flavonoids dropped precipitously after endodormancy release, including three flavonols (Apigenin, Kaempferol, and Quercetin), one flavanol glycoside (Epicatechin-glucoside), and two PAs (Procyanidins B1 and B2, Procyanidin B3) [35]. In black currant buds, flavanols exhibited two opposite trends during dormancy to bud break, with EGC and EC decreased and catechin (C) increased significantly [36]. In thidizauron-induced apple bud dormancy break, rutin and EC contents were reduced rather than increasing [37]. Similarly, during forced conifer bud break, EC content was also reduced [27]. In this study, 20 flavonoids in tea shoot buds were significantly altered by removing the axillary floral buds (Table 1). Based on their changing directions, these flavonoids can be classified into two groups. The first group exhibited higher contents in paradormant shoot buds, then decreased significantly at 1 DAF. This group includes three flavone glycosides (Apigenin 6-C-glucoside 8-C-arabinoside, C-diglucosylapigenin, and prunin) and four PAs (Procyanidin trimer isomer 1, Galloylprocyanidin dimer, Procyanidin trimer, and Procyanidin trimer isomer 4) (Table 1). We tend to believe that these flavonoids play roles in paradormancy maintenance in tea axillary shoot buds. Apigenin 6-C-glucoside 8-C-arabinoside could reduce gluconeogenesis [38]. C-diglucosylapigenin has the activity to reduce glucose levels [39]. Prunin interacts with various cyclins and cyclin-dependent kinases to impact cell cycle regulation [40,41]. PAs and anthocyanin have been implicated in tomato seed dormancy [42]. In *Arabidopsis tt12* mutant, reduced testa PA deposition is associated with reduced seed dormancy [43]. PAs have the ability to bind proteins and regulate auxin transport [44]. In addition, PAs are toxic to fungi, bacteria, and insects [45] and may act as a mechanical barrier to reduce water uptake, thus keeping axillary shoot buds at lower water status [46]. Thus, elevated PAs could regulate hormone metabolism and water contents to facilitate paradormancy set. In contrast, the second group showed lower contents in paradormant shoot buds, then increased significantly at 1 or 3 DAF. This group includes Tricetin, Rutin, Quercetin 3-O-glucosyl rutinoside, Gallocatechin 3′-O-gallate, Epigallocatechin gallate, Epigallocatechin 1, and Prodelphinidin A2 3′-gallate (Table 1). We speculate that this group of flavonoids is likely associated with paradormancy release and bud growth.

Interestingly, three flavone glycosides all accumulated in paradormant shoot buds; this was in sharp contrast with Flavonol glycoside, Linalool primeveroside isomer 1, and Phenylethyl primeveroside isomer 1 (Table 1). It remains unclear why flavone glycosides were specifically accumulated in view of the general reduced flux of phenylpropanoid pathway and flavonoid pathway in the paradormant shoot buds (Table 1; Figure S3). Buer et al. (2007) demonstrated that naringenin, the precursor for flavone synthesis, can travel long distances via cell-to-cell movement to distal tissues [47]. This raises the possibility that the growing axillary floral buds could export naringenin to their local axillary shoot buds. This scenario could provide a potential mechanism for how the growing axillary floral buds manipulate their local axillary shoot bud metabolism for paradormancy set.

It is worth mentioning that many metabolites, such as flavonoids [48,49], sugar and sugar alcohols (trehalose, raffinose, galactinol) [23,50,51,52], α-tocopherol, and theobromine [53], also have potent antioxidative activities. In addition, they possess diverse scavenge activities toward various forms of ROS. Thus, their reconfigurations during paradormancy-to-growth transition are expected to alter shoot bud meristem ROS niches, which have been implicated in cell division and differentiation regulation [54,55,56,57].

## 4. Materials and Methods

### 4.1. Plant Material

*Camellia sinensis* cv. Huangdan was grown in the tea garden located in Fujian Agriculture and Forestry University campus (Fuzhou, China; 26°08′19″ N, 119°24′06″ E) for 25 years. In August, the axillary shoot bud and the axillary floral buds emerged from the same leaf nodes. The axillary shoot buds initiate expansion as local axillary floral buds, then slow down, eventually coming to complete suspension, while the growth of the axillary floral buds continues. At this stage, the majority of the axillary shoot buds are similar in size. We noticed that the axillary shoot bud growth suspension is highly coordinated with the axillary floral bud development, which shows an asynchronous pattern. The paradormant axillary shoot buds are selected based on following criteria: (1) its appearance and uniform size; (2) the developmental stage of the local axillary floral buds. On 30 August 2016, the axillary floral buds were carefully removed from selected leaf nodes and the axillary shoot buds were kept intact. The axillary shoot buds were then harvested on 31 August (HD_1 samples) and on 2 September (HD_3 samples). Some axillary shoot buds were harvested on 30 August right before floral bud removal as a control (HD_0 sample).

### 4.2. Metabolite Extraction

A total of ~40 mg axillary shoot buds was weighed into a 1.5 mL centrifuge tube, ground into powder with a plastic pestle in the presence of liquid nitrogen, and 200 μL of 80% (*v*/*v*) methanol (chromatographic grade) solution was added and ultra-sonicated for 30 min under 25 °C, then centrifuged at 12,000× *g* for 15 min (25 °C). The supernatant was collected in a 1.5 mL tube. The pellet was re-extracted two more times as above; the supernatant was pooled. Then, the pellet was re-extracted three more times by 200 μL of 80% methanol containing 0.1% (*v*/*v*) formic acid as extraction solvent, and the supernatant was pooled, then filtered through 0.22 μm membrane. The filtrate was divided into two parts for UPLC-QTOF MS and GC-TOF MS analysis, respectively. For UPLC-QTOF MS analysis, the filtrate was diluted 50 times with 80% methanol/0.1% (*v*/*v*) formic acid solution. For GC-TOF MS analysis, 500 μL filtrate aliquot was dried down completely in CentriVap Console (Labconco, KS, USA), 80 μL methoxyl amine (20 mg/mL) in pyridine was added and sonicated to dissolve the pellet, kept at room temperature for 30 min, then 80 μL N-methyl-N-(trimethylsiyl) trifluoroacetamide (MSTFA) plus 1% (*v*/*v*) trimethylchlorosilane (TMCS) was added, placed in a 70 °C oven for 60 min, then left at room temperature in the dark for an additional 2 h. A total of 160 μL pyridine was added to the derivitized sample before sample injection [58].

### 4.3. UPLC-QTOF MS Analysis

Tea metabolite was analyzed by Waters Acquity ultra-performance liquid chromatography coupled to a SYNAPT G2-Si HDMS QTOF mass spectrometry (Waters, Manchester, UK). One μL of sample was injected into Acquity UPLC HSS T3 column (100 × 2.1 mm, 1.8 μm, Waters, Manchester, UK) for chromatographic separation. The mobile phases A and B were water/0.1% (*v*/*v*) formic acid and acetonitrile/0.1% (*v*/*v*) formic acid, respectively. The flow rate was 0.3 mL min^−1^ with linear elution gradient: 0 min, 1% B; 2 min, 7% B; 13 min, 40% B; 14 min, 99% B; 18 min, 99% B; 18.1 min, 1% B; held for 5 min. Each sample was analyzed under the positive ionization mode and the negative ionization mode, respectively. The MS was acquired in continuum mode with 10 to 40 eV collision energy. The mass parameter settings were as follows: capillary voltage, 1.5 kV (ESI^+^) and 2.5 kV (ESI^−^); ion source temperature, 120 °C; desolvation temperature, 500 °C; cone gas flow rate, 50 L/h; desolvation gas flow, 800 L/h; *m*/*z* range, 50–1200 Da [59]. The instrument was operated by MassLynx software (version 4.1, Waters, Milford, MA, USA). UPLC-MS data were processed by Progenesis QI software (v2.1, Nonlinear Dynamics, Newcastle upon Tyne, UK) with default settings; normalized peak areas were exported to Microsoft Office Excel, and outliers were manually removed. Metabolites were identified by using an in-house authentic standard spectra library and online spectral databases [60,61,62,63,64].

### 4.4. GC-TOF MS Analysis

Derivitized samples were analyzed using an Agilent 7890B gas chromatography (Agilent, Santa Clara, CA, USA) coupled with a Pegasus HT time-of-flight mass spectrometer (LECO, Saint Joseph, MI, USA). One μL of sample was injected into capillary column (Rxi-5Sil, 30 m × 0.25 mm × 0.25 μm, Restek, Bellefonte, PA, USA); helium was used as the carrier gas with a flow rate of 1 mL min^−1^. The temperature settings were as follows: injector, 270 °C; transmission line, 275 °C; ion source, 250 °C. The oven was initiated at 80 °C with 5 min solvent delay, then ramped at 5 °C min^−1^ to 310 °C, and held for 6 min. The electron collision energy was −70 eV, and the detector voltage was 1440 V. The scan range was 45–600 atomic mass unit (AMU) with acquisition rate of 10 spectra s^−1^. Raw data were processed by Chroma TOF software (ver 4.51.6, LECO, St. Joseph, MI, USA). Metabolites were identified by quest NIST library or compared to authentic standards, and the mean area of the selected characteristic ions was used for quantification.

### 4.5. Statistical Analysis

MS peak areas were normalized to sample weight. Partial least squares discriminate analysis (PLS-DA) was performed by using SIMCA-P+ 14.1 software (Umetrics, Umeå, Sweden); one-way ANOVA analysis was conducted by using SPSS software (V25, IBM, Armonk, NY, USA).

## Figures and Tables

**Table 1:** Tea shoot bud metabolites that were significantly altered by removing the axillary floral buds.

Compounds	Platform		Peak Area	
		HD_0	HD_1	HD_3
Sugars and sugar alcohols				
Glucose-1-phosphate	GC–MS	4735 ± 1167 ^b^	15,016 ± 2191 ^a^	16,453 ± 1388 ^a^
Fructose 1	GC–MS	ND ^b^	37,175 ± 5760 ^a^	26,315 ± 6877 ^a^
D-(-)-Tagatofuranose	GC–MS	109,341 ± 19,127 ^b^	213,012 ± 3111 ^a^	191,165 ± 11,631 ^a^
Trehalose	GC–MS	143,896 ± 16,715 ^a^	99,136 ± 20,320 ^ab^	56,627 ± 21,021 ^b^
Raffinose	GC–MS	15,683,254 ± 1,873,902 ^a^	7,252,704 ± 317,388 ^b^	8,417,784 ± 3,488,121 ^ab^
Galactinol	GC–MS	2,887,986 ± 325,887 ^a^	2,465,573 ± 563,001 ^ab^	1,242,611 ± 394,221 ^b^
α-D-Xylopyranose	GC–MS	16,952 ± 111 ^a^	ND ^b^	15,533 ± 3206 ^a^
Amino acids and amins				
Asparagine 4	GC–MS	572,463 ± 112,880 ^a^	308,529 ± 113,548 ^ab^	104,202 ± 48,827 ^b^
N,N,O-Triacetylhydroxylamine	GC–MS	ND ^c^	7545 ± 52 ^b^	14,886 ± 21 ^a^
Benzenamine	GC–MS	10,604 ± 70 ^b^	12,449 ± 85 ^b^	18,189 ± 2730 ^a^
Organic acids and phenolic acids				
Arabinonic acid	[M + H]+	1448 ± 36 ^b^	588 ± 53 ^a^	703 ± 112 ^a^
Malonic acid	GC–MS	46,286 ± 9803 ^b^	84,527 ± 13361 ^b^	149,950 ± 19002 ^a^
5-p-coumaroylquinic acid	[M − H]−	237 ± 15 ^b^	491 ± 84 ^ab^	1289 ± 447 ^a^
Benzoic acid 1	GC–MS	13,645 ± 1392 ^b^	15,410 ± 2558 ^b^	23,169 ± 1098 ^a^
Benzoic acid 2	GC–MS	262,832 ± 4059 ^a^	194,220 ± 26,305 ^b^	265,754 ± 379 ^a^
5-Ethoxy-3,4-dihydro-2H-pyrrole-2-carboxylic acid	GC–MS	ND ^b^	ND ^b^	7572 ± 825 ^a^
Flavones and flavone glycosides				
Tricetin	[M + H]+	1402 ± 170 ^b^	2101 ± 124 ^a^	1607 ± 129 ^b^
C-diglucosylapigenin	[M − H]−	748 ± 79 ^a^	513 ± 55 ^b^	513 ± 11 ^b^
C-diglucosylapigenin	[M + H]+	990 ± 748 ^a^	680 ± 61 ^b^	636 ± 8 ^b^
Apigenin 6-C-glucoside 8-C-arabinoside	[M − H]−	1952 ± 116 ^a^	1515 ± 139 ^b^	1193 ± 116 ^b^
Apigenin 6-C-glucoside 8-C-arabinoside	[M + H]+	2889 ± 300 ^a^	2159 ± 270 ^ab^	1786 ± 176 ^b^
Prunin	GC–MS	14,725 ± 14,420 ^a^	10,156 ± 1027 ^b^	11,844 ± 659 ^ab^
Flavonol glycosides				
Rutin	[M − H]−	2142 ± 58 ^b^	3779 ± 261 ^a^	2536 ± 111 ^b^
Rutin	[M + H]+	893 ± 25 ^b^	1619 ± 141 ^a^	1157 ± 48 ^b^
Quercetin 3-O-glucosyl rutinoside	[M − H]−	1153 ± 173 ^b^	2138 ± 179 ^a^	1419 ± 183 ^b^
Flavanols				
Gallocatechin 3′-O-gallate	[M − H]−	252 ± 74 ^b^	713 ± 112 ^a^	1306 ± 325 ^b^
Epigallocatechin gallate	[M − H]−	1,102,389 ± 25132 ^b^	1,259,731 ± 30219 ^a^	1,131,323 ± 43874 ^b^
Epigallocatechin gallate	[M + H]+	160,946 ± 3510 ^b^	179,918 ± 1830 ^a^	166,984 ± 7311 ^b^
Epigallocatechin 1	GC–MS	74,737 ± 10,588 ^b^	132,233 ± 10,310 ^a^	93,694 ± 19,126 ^b^
Proanthocyanidins				
Procyanidin trimer isomer 1	[M − H]−	1674 ± 87 ^a^	962 ± 105 ^b^	1710 ± 257 ^a^
Procyanidin trimer isomer 1	[M + H]+	2774 ± 218 ^a^	1803 ± 214 ^b^	3029 ± 245 ^a^
Galloylprocyanidin dimer	[M − H]−	51,022 ± 3318 ^a^	42,665 ± 827 ^b^	43,313 ± 1823 ^ab^
Procyanidin trimer isomer 3	[M − H]−	511 ± 37 ^a^	262 ± 48 ^b^	542 ± 83 ^a^
Galloylated trimeric proanthocyanidin	[M − H]−	3320 ± 34 ^a^	2672 ± 137 ^b^	2349 ± 62 ^c^
Galloylprodelphinidin dimer	[M − H]−	75,967 ± 3931 ^a^	61,363 ± 2054 ^ab^	53,936 ± 8451 ^a^
Procyanidin trimer isomer 2	[M − H]−	5246 ± 296 ^ab^	3623 ± 461 ^b^	5696 ± 694 ^a^
(E)GC-(E)CG dimer	[M − H]−	50,924 ± 1577 ^a^	44,062 ± 1442 ^ab^	37,029 ± 3672 ^b^
EC-EGCG dimer	[M + H]+	30,461 ± 1037 ^a^	26,529 ± 1349 ^ab^	22,612 ± 1547 ^b^
Procyanidin trimer isomer 4	[M + H]+	2484 ± 218 ^ab^	1759 ± 197 ^b^	2727 ± 254 ^a^
Prodelphinidin A2 3′-gallate	[M + H]+	1909 ± 133 ^b^	2002 ± 122 ^b^	2528 ± 163 ^a^
Antioxidants				
α-Tocopherol	GC–MS	102,347 ± 19,412 ^b^	91,070 ± 22,521 ^b^	178,108 ± 975 ^a^
Theobromine	[M + H]+	6751 ± 1740 ^b^	28,939 ± 2641 ^a^	42,349 ± 7566 ^a^
Volatile glycosides				
Linalool primeveroside isomer 1	[M − H]−	75 ± 15 ^b^	737 ± 310 ^a^	1124 ± 301 ^a^
Phenylethyl primeveroside isomer 1	[M + Na]+	360 ± 21 ^b^	593 ± 12 ^a^	621 ± 48 ^a^
Others				
1,3-Dioxolane 1	GC–MS	34,645 ± 228 ^a^	14,556 ± 1088 ^b^	20,223 ± 4971 ^b^
1,3-Dioxolane 3	GC–MS	33,013 ± 2378 ^b^	88,752 ± 3702 ^a^	122,253 ± 23,023 ^a^
2-Methyl-3-buten-2-ol	GC–MS	105,417 ± 14,456 ^b^	228,198 ± 14,914 ^b^	430,873 ± 84,003 ^a^
2-hydroxypyridine	GC–MS	917,334 ± 112,468 ^a^	795,023 ± 73,921 ^b^	884,118 ± 123,235 ^a^

**Note:** Data are presented as mean ± standard error (*n* = 3). Statistically significant difference from the same row is denoted by different uppercase letters. ND, not detected.

## Data Availability

The original contributions presented in the study are included in the article/Supplementary Materials. Further inquiries can be directed to the corresponding author (M.C).

## Supplementary Materials

The following supporting information can be downloaded at: https://www.mdpi.com/article/10.3390/ijms26115094/s1.
