# Supplementary figures and images for: Metabolomic Analysis Provides Insights into Bud Paradormancy in Camellia sinensis cv. Huangdan
Source: Int J Mol Sci. 2025 May 26;26(11):5094. doi: 10.3390/ijms26115094 (PMC12154020; doi:10.3390/ijms26115094)

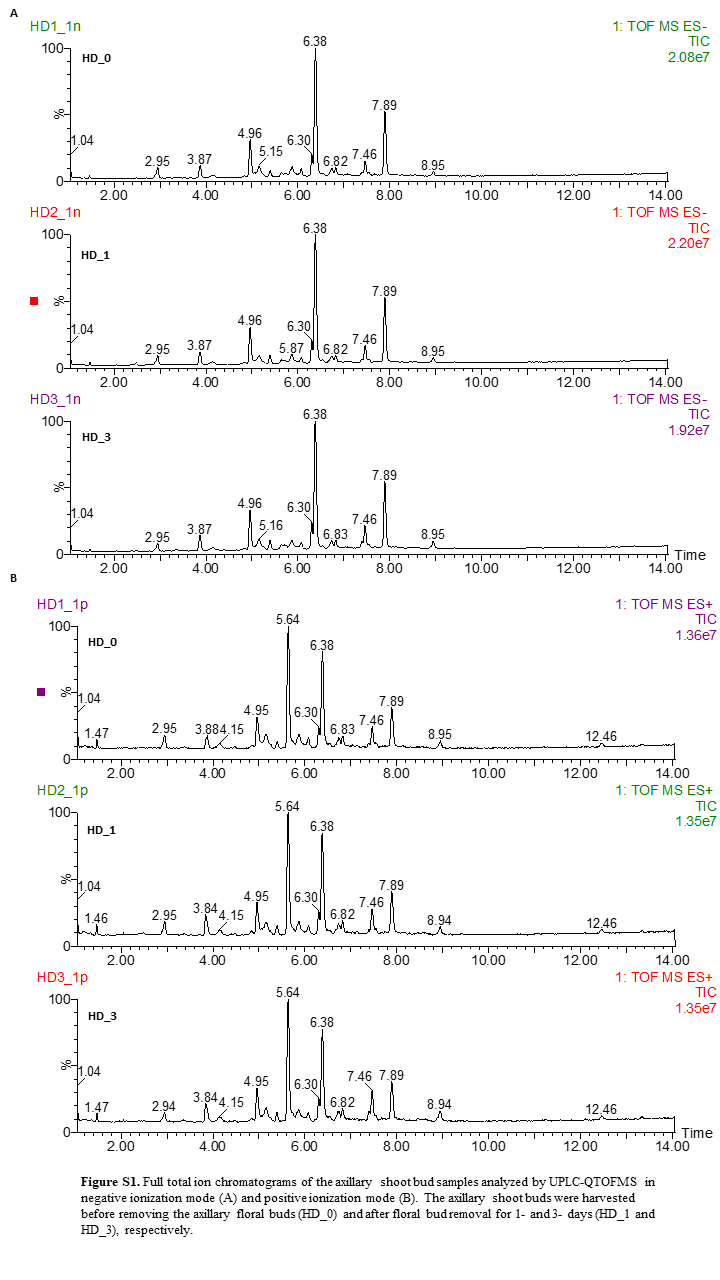

Supplement: Supplementary file 1 [file ijms-26-05094-s001.zip › Figure S1.tif]

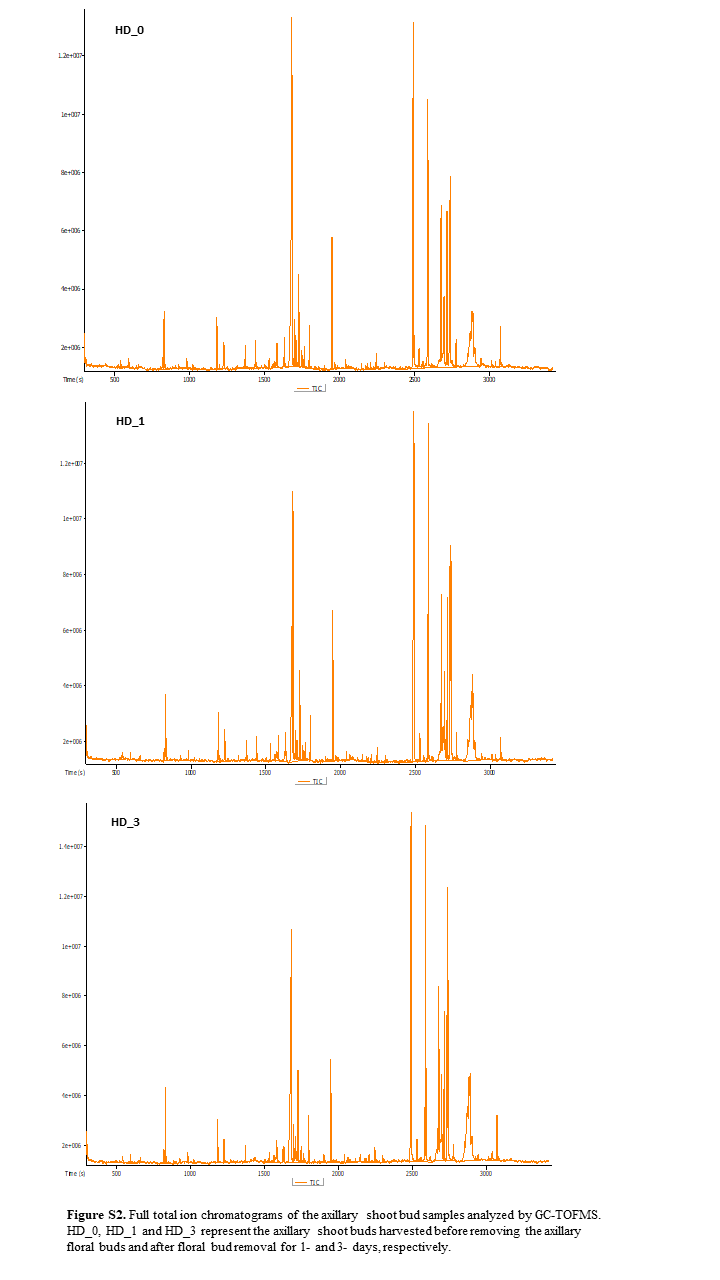

Supplement: Supplementary file 1 [file ijms-26-05094-s001.zip › Figure S2.tif]

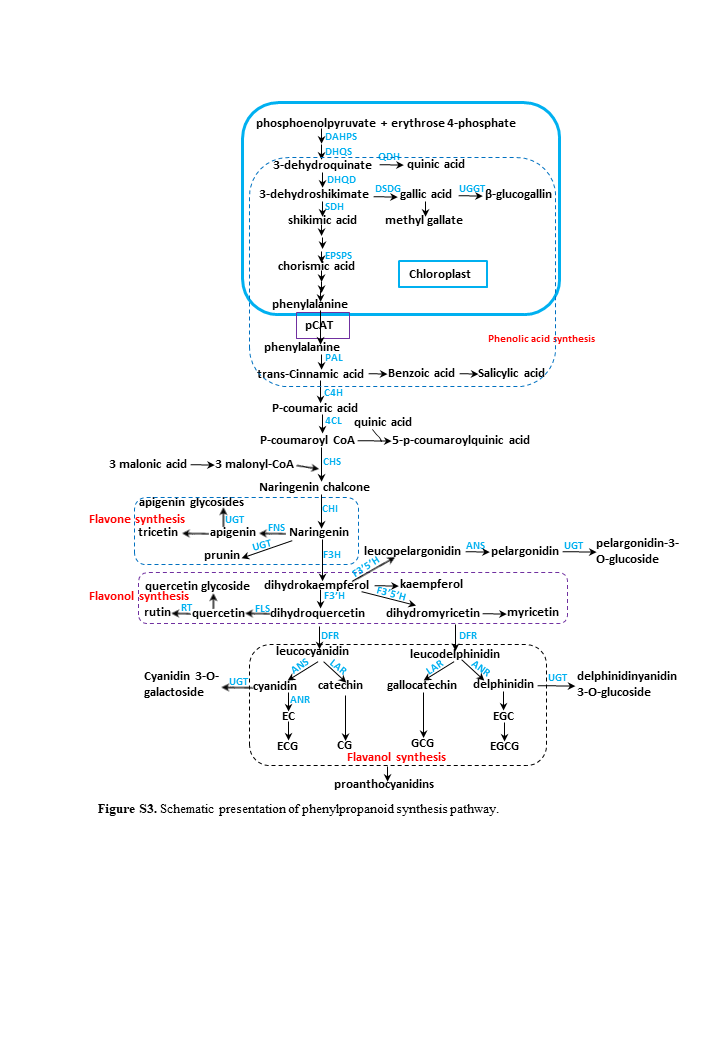

Supplement: Supplementary file 1 [file ijms-26-05094-s001.zip › Figure S3.tif]
